# Is There Synchronicity in Nitrogen Input and Output Fluxes at the Noland Divide Watershed, a Small N-Saturated Forested Catchment in the Great Smoky Mountains National Park?

**DOI:** 10.1100/tsw.2001.384

**Published:** 2001-11-22

**Authors:** H. Van Miegroet, I.F. Creed, N.S. Nicholas, D.G. Tarboton, K.L. Webster, J. Shubzda, B. Robinson, J. Smoot, D. W. Johnson, S. E. Lindberg, G. Lovett, S. Nodvin, S. Moore

**Affiliations:** Department of Forest Resources, Utah State University, Logan 84322-5215, USA

**Keywords:** nitrogen, hydrology, nitrogen, cycling, mass balance, Picea rubens (red spruce), Abies fraseri (Fraser fir), acid deposition, nitrogen saturation

## Abstract

High-elevation red spruce [Picea rubens Sarg.]-Fraser fir [Abies fraseri (Pursh.) Poir] forests in the Southern Appalachians currently receive large nitrogen (N) inputs via atmospheric deposition (30 kg N ha(-1) year(-1)) but have limited N retention capacity due to a combination of stand age, heavy fir mortality caused by exotic insect infestations, and numerous gaps caused by windfalls and ice storms. This study examined the magnitude and timing of the N fluxes into, through, and out of a small, first-order catchment in the Great Smoky Mountains National Park. It also examined the role of climatic conditions in causing interannual variations in the N output signal. About half of the atmospheric N input was exported annually in the streamwater, primarily as nitrate (NO3-N). While most incoming ammonium (NH4-N) was retained in the canopy and the forest floor, the NO3-N fluxes were very dynamic in space as well as in time. There was a clear decoupling between NO3-N input and output fluxes. Atmospheric N input was greatest in the growing season while largest NO3-N losses typically occurred in the dormant season. Also, as water passed through the various catchment compartments, the NO3-N flux declined below the canopy, increased in the upper soil due to internal N mineralization and nitrification, and declined again deeper in the mineral soil due to plant uptake and microbial processing. Temperature control on N production and hydrologic control on NO3-N leaching during the growing season likely caused the observed inter-annual variation in fall peak NO3-N concentrations and N discharge rates in the stream.

## INTRODUCTION

Earlier research has indicated that nitrogen (N) dynamics in the high-elevation red spruce [*Picea rubens* Sarg.]-Fraser fir [*Abies fraseri* (Pursh.) Poir] forests in the Southern Appalachians are currently characterized by high soil N levels, high N mineralization rates, and measurable N losses below the rooting zone in the form of nitrate ($NO_3$-N)[1,2,3]. Based on intensive biogeochemical research carried out in the Great Smoky Mountains National Park (GSMNP) since the mid 1980s[4,5], spruce-fir forests in that region have been designated at an advanced stage of N saturation (stage 2, *sensu* Aber[6,7]) due to a combination of ecosystem conditions and N deposition regime[8,9]. Atmospheric N inputs to these forests, which have considerable components of wet, dry, and fog deposition estimated at 28 kg $ha^{-1}$ $year^{-1}$ during the Integrated Forest Study (IFS), rank among the highest in North America[4,10]. Limited ecosystem N retention capacity has been indicated by high soil $NO_3$-N leaching rates (10 to 20 kg N $ha^{-1}$ $year^{-1}$) and considerable streamwater exports (15 kg N $ha^{-1}$ $year^{-1}$)[4,8].

Historically, the high-elevation spruce-fir forests in the GSMNP have largely escaped logging while fires have also remained rare as a disturbance agent[11,12]. More recently, the spruce-fir forest has been impacted by an exotic insect infestation, the balsam woolly adelgid (*Adelges picea* Ratz.), causing the death of the mature Fraser fir and creating a heterogeneous forest structure with numerous gaps and a large variation in stand age, number of live and dead standing trees, and amount of coarse woody debris (CWD) on the forest floor[13,14,15,16].

An earlier paper based on a 3-year mass balance analysis of the Noland Divide Watershed (NDW)[8], a 17-ha headwater catchment near Clingman's Dome, NC, provides evidence of the N "leakiness" of the high-elevation spruce-fir ecosystem as well as its designation as stage 2 of N saturation (*sensu* Stoddard[17]), which is characterized by elevated baseflow $NO_3$-N levels and the presence of a distinct seasonal signal in the discharge $NO_3$-N concentrations. Short-term data records, however, are limited in their capability of discerning interannual climatic variations as well as longer-term trends in ecosystem condition, deposition regime, and/or or climate. All of these factors may have a significant influence on N dynamics within and $NO_3$-N export from such sensitive (limited N-retention) watersheds.

The objective of this paper is to use currently available water chemistry data (i.e., deposition, throughfall, soil, and stream solutions) that have been collected since 1986 at the NDW and at an adjacent intensive biogeochemical research plot to (1) assist us in better understanding N biogeochemistry in this high-elevation forest, (2) establish the magnitude and timing of the variation in solution chemistry, and (3) identify broad drivers for this variation. Specifically, we ask the question whether the NDW is primarily a flow-through system with the N output signal simply reflecting the N deposition; or, alternatively, whether, how, and to what extent atmospheric N input is modified at various levels within the ecosystem. If such is the case, what are the catchment-specific modifiers, and do these control valves change measurably with variations in climatic conditions?

## METHODS

### Study Site

This analysis is based on water samples collected at the NDW (35° 34' N lat., 83° 29' W long.), a small, gauged, first-order drainage located between 1650- and 1910-m elevation in the red spruce-Fraser fir forest of the GSMNP[8]; and at the nearby Tower site, an intensive nutrient cycling research site established in 1986 at 1740-m elevation as part of the IFS (Fig. 1)[4,5].

Mean annual air temperature over the past 25 years at a climate station, located about 100 m below the watershed outlet, is 8.5ºC, ranging from an average from -2ºC in January to +18ºC in July with a frost-free period from May through September[18]. Mean annual precipitation at this climate station is ~230 cm, ranging from about 150 to 300 cm in any given year (Fig. 2). Snow accounts for about 10% of the mean annual precipitation and typically covers the ground for 50 days a year[4]. During the IFS, cloud base was typically observed at 1800 m[5].

The soils are Inceptisols with spodic characteristics classified as Dystrochrepts or Haplumbrepts, underlain by Thunderhead Sandstone[4,19,20]. They have a silt loam to sandy loam texture, and are generally shallow throughout the NDW (<50-cm depth to bedrock[21]). Soils are acidic, characterized by high organic matter content and low base saturation as well as high N mineralization and nitrification capacity[1,4,22].

In 1993, fifty 20- × 20-m vegetation plots were established in the watershed, systematically spanning the elevational range. At the onset of the study, live basal area (LBA) of overstory trees (>5-cm diameter breast height [DBH]) ranged from 14.9 to 67.5 $m^2$ $ha^{-1}$. Red spruce comprised 77% of LBA at NDW while yellow birch and Fraser fir comprised 19 and 2.5%, respectively. The relative abundance of fir increased with elevation while that of yellow birch declined. Pauley et al.[14] estimated average total biomass of live overstory trees for NDW at 220 Mg $ha^{-1}$. Fraser fir accounted for 70% of the standing dead stems, indicating the large impact of the adelgid infestation[14,15]. Fir had a density of 199 live stems and 469 dead stems per hectare, red spruce a density of 363 live stems and 64 dead stems per hectare, and yellow birch had a standing density of 135 live stem per hectare and 18 dead stems per hectare[15]. In addition to the adelgid infestation, which caused the mortality of mature fir in 1980s, the research site was further impacted by two hurricanes (Hurricane Andrew in August 1992 and Hurricane Opal in October 1995) and by an ice storm in the winter of 1995, which caused downing of live and dead trees and a significant input of CWD, especially in the upper part of the catchment.

### Solution Sampling and Analysis

At the Tower site, wet, dry, and fog deposition were determined from April 1986 through March 1989 as part of the IFS. The wet

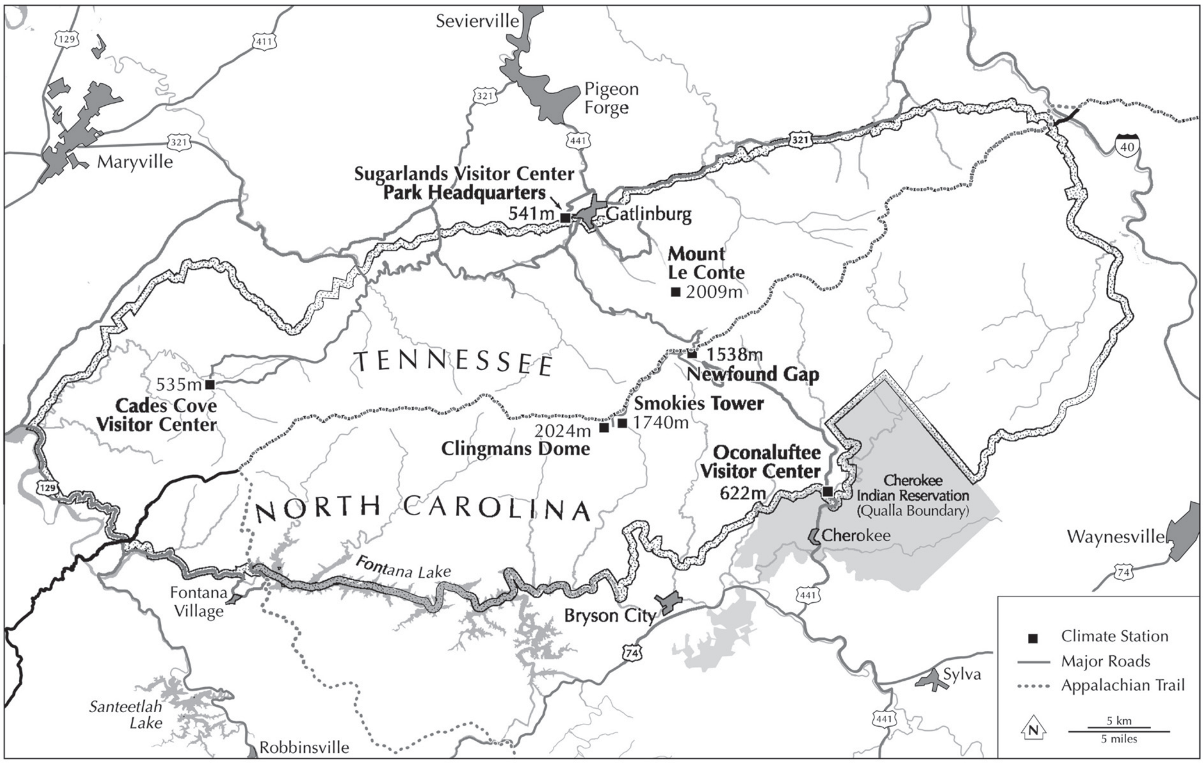

**FIGURE 1a.** Map of the Great Smoky Mountains National Park with location of the IFS Tower site, the Noland Divide Watershed, the Clingman's Dome, and the Newfound Gap climate stations.

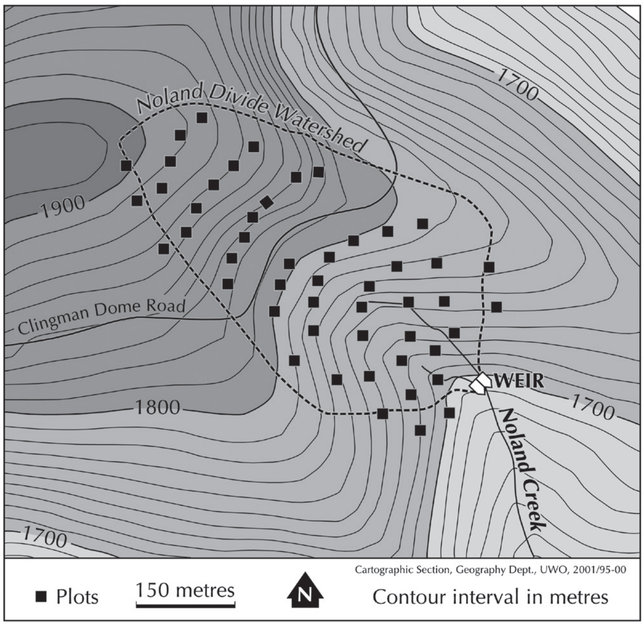

**FIGURE 1b.** Map of the Noland Divide Watershed.

fraction was derived from measurements in a forest and the dry and fog fractions were derived from measurements of atmospheric chemistry and meteorological data collected from the atmospheric tower that extended ~10 m above the surrounding forest. Collection methodologies, calculation methods, and sample analyses are detailed elsewhere[4,5,23,24]. Collection of atmospheric input was briefly interrupted until wet deposition and throughfall collection resumed in the summer of 1991 on a weekly basis.

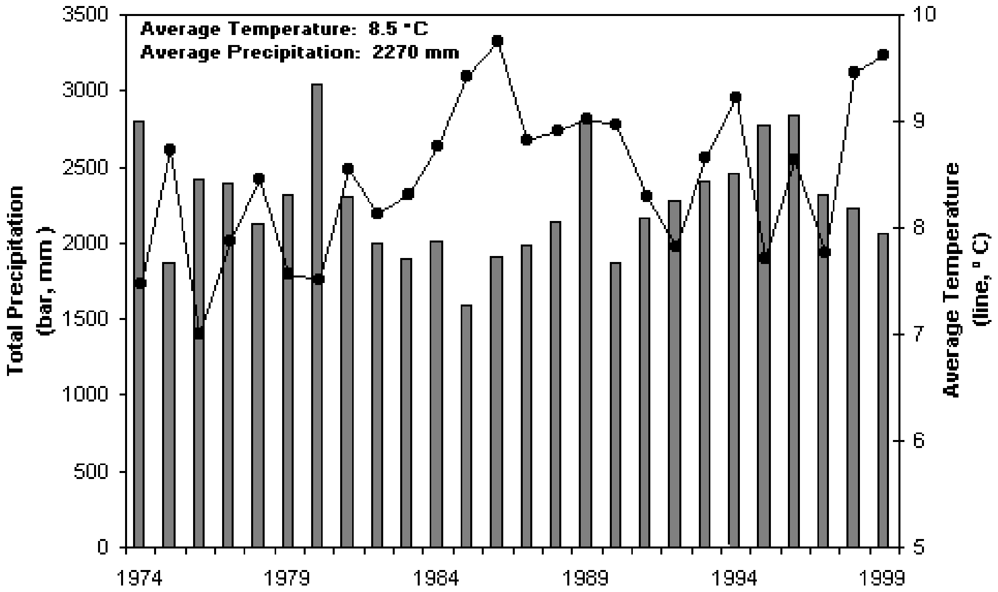

**FIGURE 2.** Annual temperature and precipitation patterns based on April through March water years based on data collected from the Newfound Gap climate station.

Wet-only deposition was collected using an Aerochemetrics automatic sampler at the original open site. During the summer months (May through October), throughfall was collected at eight random locations within the site using 1-l polyethylene bottles with 3.5-cm-diameter polyethylene funnels, and volume composited into one sample per collection period. During the winter months (November through April), the throughfall samples were collected in two large-diameter, plastic-lined buckets located on platforms ~1 m from the ground and volume composited. Precipitation and throughfall volumes were measured using wedge-type rain gauges located adjacent to each collector during the summer or by weighing the collection buckets during the winter periods. Sample methodologies followed earlier IFS protocols. All samples were analyzed for pH and conductivity immediately upon return from the field, preserved with chloroform (10 µl/30 ml of sample), and stored at 4°C until analyses of major cations and anions were performed.

Soil solutions were collected continuously underneath the forest floor and at approximately 10- and 40-cm depth in the mineral soil with fritted, glass-tension lysimeters installed at three depths. A continuous tension of approximately 10 kPa was applied by a hanging column system[4]. From 1986 through 1992, monthly samples from the four replicate lysimeters per depth were analyzed separately (n = 12); in subsequent years solutions were composited into one monthly sample per soil depth prior to analysis. Collected volumes per lysimeter were recorded and used to calculate volume-weighted average annual concentrations for a given soil depth. There were some gaps in the lysimeter data due to loss of samples prior to completion of the chemical analysis (1991 and 1992) or occasional equipment malfunction. Annual N flux at a given soil depth was obtained by multiplying the volume-weighted average concentration of $NH_4$-N or $NO_3$-N for a given depth by the annual water flux through the soil, calculated as precipitation minus potential evapotranspiration using the Thornthwaite method[25]. Precipitation and temperature values used in this method were derived from the climate station at Newfound Gap in the GSMNP (Fig. 1a).

In the summer of 1991, 1-m H-flumes were installed on the two streamlets draining the NDW (Fig. 1b). Every 15 min, height was measured, converted the measurement to a flow rate with a rating formula, and logged the data to a Campbell datalogger along with pH, conductivity, and temperature measured from Hydrolab units located in each streamlet. The data were downloaded weekly and stored in spreadsheets. Weekly grab samples were taken from both streamlets and brought back to the University of Tennessee where they were analyzed for pH, conductivity, acid neutralizing capacity, and major anions and cations including $NO_3$-N and $NH_4$-N by ion chromatography. Quality assurance/control for $NO_3$-N and $NH_4$-N were found to be acceptable based on results of interlaboratory comparison analyses, blanks, duplicate samples, and standards. The stream discharge measurements were periodically checked by manually measuring the flow and checking it against the rating curve. Daily streamwater concentrations of $NH_4$-N and $NO_3$-N were estimated by linear interpolation between the weekly samplings. These values were then averaged for both weirs, multiplied by the discharge rates, and expressed on a per hectare basis by dividing by the catchment area.

All N flux analyses were based on water years extending from April through March of the subsequent year as was done during the IFS[5]. Monthly N flux was calculated as the sum of the available (daily, weekly, or biweekly) N fluxes during that month, and similarly, the annual N flux was calculated as the sum of the monthly N fluxes. While atmospheric N input as wet, dry, and fog deposition was determined for $NH_4$-N, $NO_3$-N, and total N during the IFS period (1986 to 1989), only wet deposition of inorganic N species was measured at the Tower site in subsequent years. Total inorganic N deposition was obtained by multiplying wet deposition by a factor of 4.4 and the separation into dry, wet, and fog for $NH_4$-N and $NO_3$-N was based on the ratios derived during the 1986 to 1989 period: $NH_4$-N:$NO_3$-N = 36:64 (total 100) for total deposition; Dry:Wet:Fog = 6:11:19 (total 36) for $NH_4$-N and 39:12:13 (total 64) for $NO_3$-N[5]. In the past, water samples were not analyzed for dissolved organic N (DON), and consequently our N budgets and N flux calculations for the NDW did not consider this N form.

Statistical significance of the differences in N fluxes among the solutions was tested only on data from a common collection period (April 1993 to March 1997) using a nonparametric Kruskal-Wallis one-way analysis of variance on ranked data followed by Tukey pairwise multiple comparison tests as the data failed the test for normality (SIGMASTAT 2.03, SPSS Inc.).

## RESULTS AND DISCUSSION

Based on the simple atmospheric input-streamwater output N-mass balance presented in Table 1, approximately 50% the incoming N was retained at NDW. On average about 30 kg $ha^{-1}$ was deposited annually to the NDW while only 15 kg $ha^{-1}$ $year^{-1}$ was discharged in the stream, mostly in the form of $NO_3$-N. The retention capacity of this spruce-fir ecosystem was low compared with N retention typically observed in North American forests, especially when they are N-limited[26]. The incoming inorganic N flux consisted of one-third $NH_4$-N, but $NH_4$-N export losses were consistently low (<1 kg $ha^{-1}$ $year^{-1}$), indicating almost complete retention of this form. While the annual N budget would suggest that this catchment was a simple flow-through system especially for $NO_3$-N, a somewhat different picture emerged when examining the changes in N fluxes as the solution passed through the various components of the watershed (Table 2).

The forest canopy acted as the first filter of incoming N, retaining nearly 70% of the atmospheric $NH_4$-N deposition and about 30% of the atmospheric $NO_3$-N deposition. Despite this apparent retention of N in the canopy, differences between above- and below-canopy N fluxes were not statistically significant for either $NH_4$-N or $NO_3$-N ($p < 0.05$). Our results were consistent with earlier observations of inorganic N retention by the forest canopy at the Tower site and at NDW[10,27]. Canopy uptake of inorganic N has been reported in many North American forests[5,10], but the reported magnitude of this uptake seems to be influenced by the method of estimating N deposition[4]. In terms of ecosystem N biogeochemistry, canopy N retention is important because it (1) can cause a considerable lag period between the time of atmospheric deposition and the availability of N in the soil; (2) modifies the form of N input to the soil, i.e., from a dissolved hydrologic flux to particulate litterfall flux or from an inorganic to organic N form[10]; and (3) reduces the vegetation's reliance on N derived from the mineral soil, thus modifying the belowground N retention capacity.

Once the solution reached the forest soil, $NH_4$-N fluxes became progressively smaller with the greatest removal in the forest floor (83% of incoming flux) and the upper mineral soil (an additional 60% of the $NH_4$-N leaching from the forest floor). Annual $NH_4$-N losses below the rooting zone and via stream discharge were significantly lower than the input flux (<1 vs. 12 kg $ha^{-1}$ $year^{-1}$; Table 2). The removal of $NH_4$-N from the percolating solution may have resulted from biological immobilization or, more likely, from the microbial conversion into $NO_3$-N, given the typically high nitrification capacity of these soils[1,4,22,28].

The $NO_3$-N fluxes were more dynamic. High rates of mineralization and subsequent nitrification of the large soil organic N pool accounted for the significant increase in $NO_3$-N flux at the interface between the forest floor and A-horizon (from 18 kg N $ha^{-1}$ $year^{-1}$ via throughfall to 37 kg $ha^{-1}$ $year^{-1}$ below the forest floor). The magnitude of this internal source of $NO_3$-N (on average about 20 kg $ha^{-1}$ $year^{-1}$) was similar to the external (atmospheric) source of $NO_3$-N. The release of $NO_3$-N in the upper soil was highly variable among individual years as indicated by the large coefficient of variation (48%), such that at the lower end of the range, the catchment indeed acted as a simple flow-through system. In years corresponding to the higher extreme, the vertical N flux profile was more complex, with a major release of $NO_3$-N near the surface followed by its partial removal at greater soil depth, possibly reflecting root uptake. This is supported by recent overstory N uptake calculations for the NDW indicating an average annual N sequestration of 8 kg $ha^{-1}$ $year^{-1}$ in aboveground biomass increment of the spruce-fir forest[29]. Values throughout the catchment ranged from 3 to 13 kg $ha^{-1}$ $year^{-1}$ depending on the stand structure and the relative proportion of dead fir. The Tower site, where the soil solutions were collected, was located at a lower elevation range of the NDW

**TABLE 1**
**Average Annual Inorganic N Input and Output Fluxes at the NDW (average ± standard deviation) from April 1993 through March 1997**

| | | Input (kg $ha^{-1}$ $year^{-1}$) | | | Output (kg $ha^{-1}$ $year^{-1}$) |
|---|---|---|---|---|---|
| **Inorg. N** | | 32.2 ± 6.61 *(29.4 ± 5.5)*† | | | 15.0 ± 1.84 *(15.1 ± 1.62)*§ |
| **Speciation** | **Total** | **Dry** | **Wet** | **Fog** | |
| $NH_4$-N | 11.6 *(10.6)* | 2.0 *(1.8)* | 3.6 *(3.3)* | 6.0 *(5.5)* | 0.2 ± 0.1 *(0.2 ± 0.11)* |
| $NO_3$-N | 20.6 *(18.8)* | 12.5 *(11.4)* | 3.8 *(3.5)* | 4.3 *(4.0)* | 14.8 ± 1.75 *(14.9 ± 1.56)* |

† Italiced N inputs represent averages for all input data available for periods April 1986 – March 1989 and April 1992 – March 1997.

§ Italiced N outputs represent averages for all output data available for period April 1992 – March 1998.

**TABLE 2**
**Average Annual Inorganic N Fluxes at Different Levels in the NDW (average, standard deviation and coefficient of variation) from April 1993 through March 1997**

| | $NH_4$-N (kg ha$^{-1}$ year$^{-1}$) | | | |
|---|---|---|---|---|
| **Watershed Level** | **Average** | **Std. Dev.** | **CV (%)** | **N Release Index**† |
| Above Canopy | 11.6 *(10.6)*§ **a**‡ | 2.38 *(2.40)* | 20 *(23)* | |
| Below canopy | 3.4 *(3.0)* **ab** | 0.65 *(0.93)* | 19 *(30)* | 0.29 *(0.29)* |
| Below forest floor | 0.6 *(0.7)* **ab** | 0.14 *(0.30)* | 24 *(45)* | 0.17 *(0.22)* |
| Soil – 10 cm | 0.2 *(0.2)* **b** | 0.17 *(0.14)* | 77 *(61)* | 0.38 *(0.35)* |
| Soil – 40cm | 0.2 *(0.3)* **b** | 0.24 *(0.25)* | 121 *(93)* | 0.89 *(1.18)* |
| Stream | 0.2 *(0.2)* **b** | 0.11 *(0.11)* | 46 *(58)* | 1.20 *(0.70)* |

| | $NO_3$-N (kg ha$^{-1}$ year$^{-1}$)[a] | | | |
|---|---|---|---|---|
| **Watershed Level** | **Average** | **Std. Dev.** | **CV (%)** | **N Release Index**† |
| Above canopy | 20.6 *(18.8)* **ab** | 4.23 *(3.43)* | 20 *(18)* | |
| Below canopy | 14.9 *(13.3)* **b** | 2.42 *(4.07)* | 16 *(31)* | 0.72 *(0.71)* |
| Below forest floor | 36.8 *(33.3)* **a** | 17.66 *(14.14)* | 48 *(42)* | 2.47 *(2.50)* |
| Soil – 10 cm | 19.7 *(17.4)* **ab** | 2.48 *(3.94)* | 13 *(23)* | 0.54 *(0.52)* |
| Soil – 40 cm | 23.3 *(20.7)* **ab** | 4.53 *(4.66)* | 19 *(22)* | 1.18 *(1.19)* |
| Stream | 14.8 *(14.9)* **b** | 1.75 *(1.56)* | 12 *(10)* | 0.63 *(0.72)* |

† N Release Index from each watershed component is calculated as $N_{output}/N_{input}$, with $N_{output}$ = flux at a given level in the watershed; $N_{input}$ = flux at previous level in the watershed.

§ Italicized values represent averages calculated from all available data. For the various fluxes the periods are: above and below canopy solutions (1986–1988, 1992–1996); soil solutions below the forest floor and at 10- and 40-cm soil depth (1986–1989, 1993–1996); streamwater (1992–1997).

‡ Averages followed by different letters indicate statistical difference between level ($p < 0.05$; Tukey Test) based on data from the 1993–1996 water years.

and may thus fall at the higher end of the N uptake values as fir did not constitute a significant overstory component. Furthermore, most N uptake calculations do not typically include understory dynamics. Jamison et al., in a study at another high-elevation site in the GSMNP near Mt LeConte, found significantly lower $NO_3$-N concentrations in the soil solution under spruce-fir forest where significant regeneration was present irrespective of the overstory canopy condition[30]. Consequently, both overstory and understory N requirements have the capacity to attenuate the $NO_3$-N flux through the soil, but only during the period of active growth. We have observed but not yet quantified significant spruce and fir regeneration in some parts of the NDW. Studies are currently underway to elucidate the role of understory regeneration, and particularly of tree regeneration, in N biogeochemistry, as there are indications that streamwater $NO_3$-N levels have been decreasing without a concomitant decline in N deposition.

Overall, on average about 10 kg ha$^{-1}$ year$^{-1}$ more $NO_3$-N leached out of the rooting zone than entered via throughfall. However, this excess $NO_3$-N was subsequently converted or removed from the subsurface solution prior to it entering the stream such that there was no significant difference between the throughfall and stream fluxes. The mechanism of this $NO_3$-N loss is yet unknown, but high $NO_3$-N levels coupled with high organic matter contents and largely wet soil conditions represent ideal conditions for denitrification.

Even if this was not a simple flow-through system, was there at least some synchronicity between N input and $NO_3$-N outputs in this catchment? In addition to a modification in the magnitude of the N flux as it passed through the system, there was also a significant shift in the timing of $NO_3$-N output relative to the N input. In Fig. 3 we examined the seasonal distribution of water and N input-output fluxes in the catchment based on multiyear averages. While there was measurable precipitation throughout the year, precipitation and discharge maxima at the NDW generally coincided with the dormant season, particularly from January to March (Fig. 3a). Lower discharge rates relative to precipitation rates during the growing season reflected the influence of significant evapotranspiration losses in late spring and summer. Monthly atmospheric N deposition did not follow the seasonal precipitation pattern. The majority of total inorganic N was deposited in the catchment during the growing season (Fig. 3b), possibly due to higher dry deposition input since wet deposition tends to closely follow water fluxes[31]. The N input flux was high from April to September, lower from October to December, and higher again from January to March. In contrast, most $NO_3$-N was exported from the catchment during the dormant season, from November through March, with the highest monthly $NO_3$-N fluxes in winter (January to March). Thus, there appeared to be general temporal decoupling between N inputs and N losses from the system.

How then, did individual years behave in terms of timing and magnitude of $NO_3$-N outputs relative to N inputs to the soil? Were patterns consistent, and if not, could this differential response be explained logically? Our next step in the data analysis was to more closely examine the relationship between monthly $NO_3$-N stream discharge and throughfall N input for the four water years for which solution data at all catchment levels were available (Fig. 4). In these graphs, synchronicity between N input and output would be indicated by a linear relationship between the monthly fluxes (i.e., an increase in throughfall would be followed by as proportional increase N output). Points aligning along the 1:1 line would imply a simple flow-through system (output = input); points located above the 1:1 line indicated net release (output > input) in that month; and fluxes below the line indi-

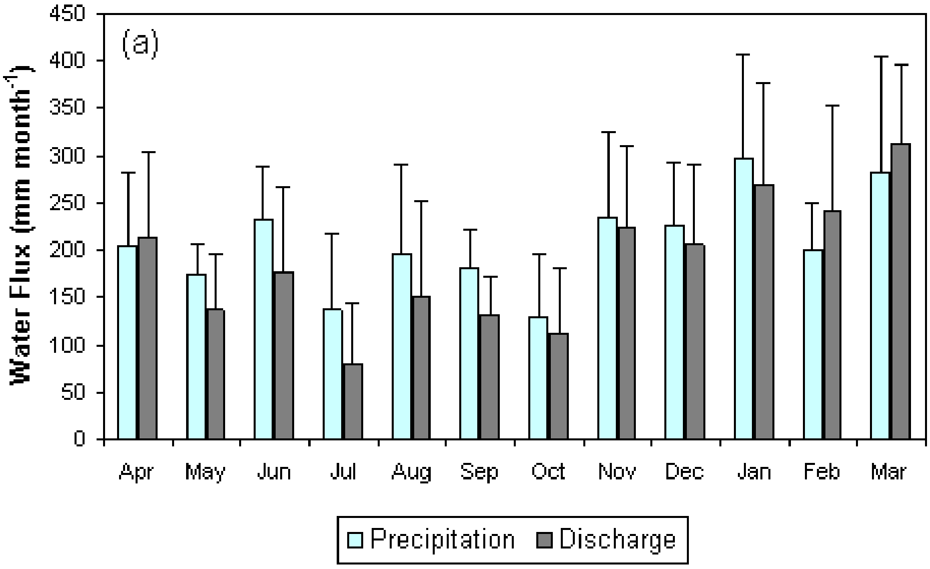

**FIGURE 3a.** Monthly distribution of water and N fluxes (average ± standard deviation) based on data from April 1992 to March 1998: precipitation and discharge (mm $month^{-1}$)

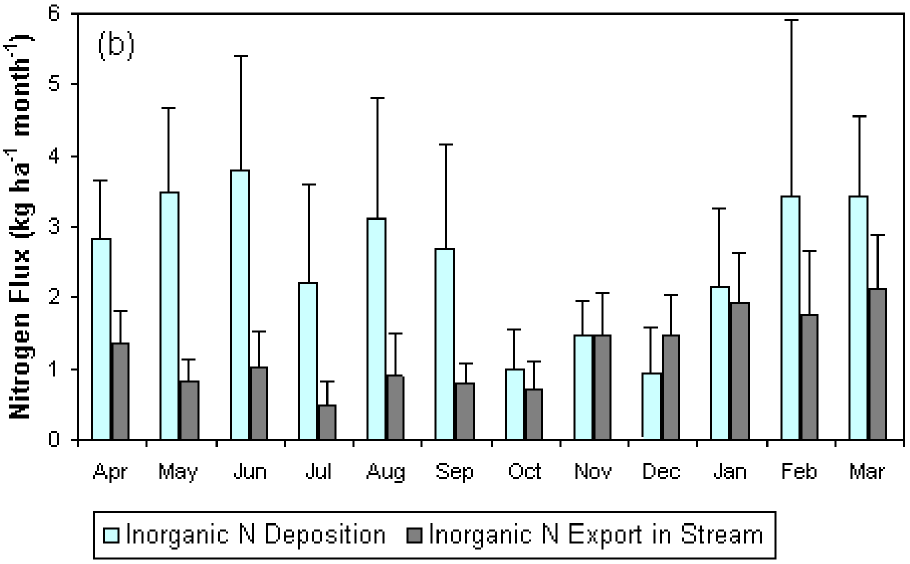

**FIGURE 3b.** Monthly distribution of water and N fluxes (average ± standard deviation) based on data from April 1992 to March 1998: atmospheric N deposition and stream N export (kg $ha^{-1}$ $month^{-1}$).

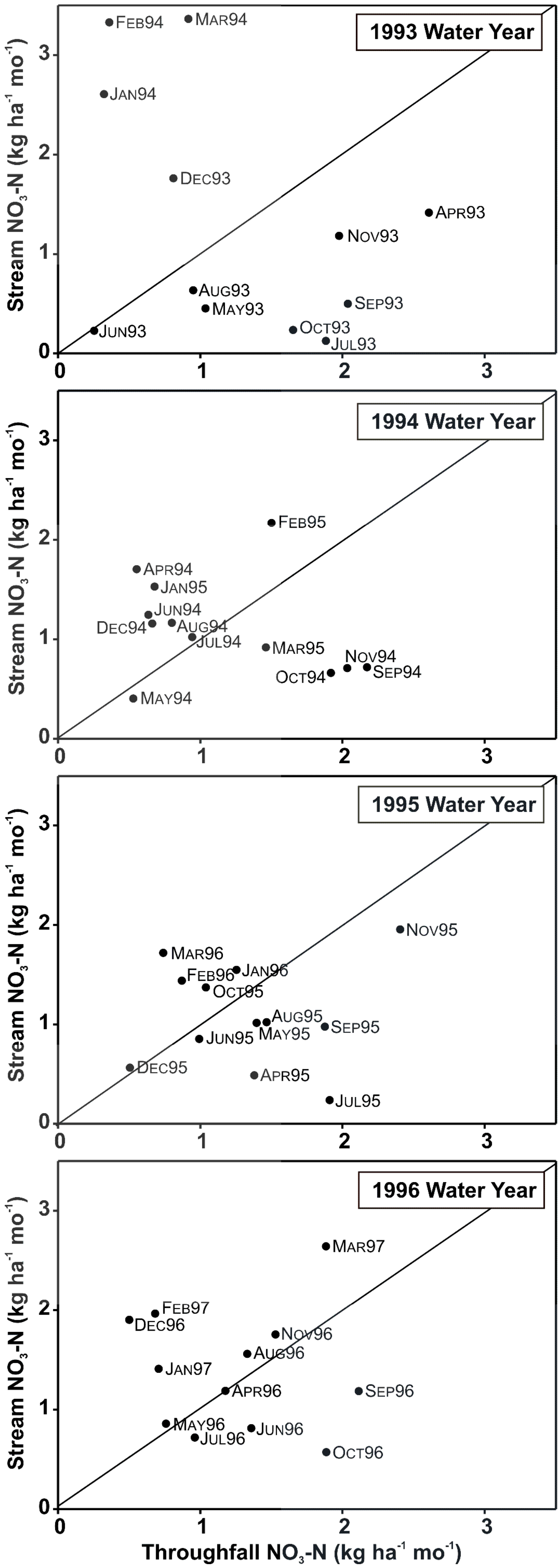

**FIGURE 4.** Relationship of total monthly $NO_3$-N input vs. N output flux for 4 water years.

cated net retention of the incoming N (output < input) or loss due to denitrification.

There was no detectable linear relationship between monthly throughfall N input and streamwater $NO_3$-N export in any of the years, further substantiating the lack of synchronicity between N input and output fluxes in this catchment (Fig. 3). Furthermore, individual years showed variable responses. The 1993 water year (April 1993 to March 1994) exemplified the distinct separation between growing season and dormant season in terms of N processing (Fig. 4a). Substantial net N retention ($NO_3$-N stream export < N throughfall input) occurred throughout the growing season and into the fall (through November 1993), while substantial net N release from the catchment ($NO_3$-N stream export > N throughfall input) was limited to the winter period (December 1993 to March 1994). These winter months typically showed net N release in all the water years. In the other 3 years, the seasonal shift from net N retention to N release was still present but attenuated. In the 1994 and 1996 water years, the growing season N retention capacity of the system was substantially lower, indicated by a shift of the growing season fluxes towards the 1:1 line (Fig. 4b and 4d). This lowered net N retention was particularly noticeable in 1994 (Fig. 4b) where some months in the growing season (April, June, August) actually showed net N release while in two months (May, July) N input and output were approximately in balance. The N dynamics in the 1995 water year were different still. The shift towards net N retention in the growing season was still evident, but the transition to net N release in the dormant season was much less pronounced (Fig. 4c). While the lack of net N release may be real, this particular water year was distinctive in that some streamwater $NO_3$-N concentration data were missing for the period October to March, perhaps resulting in an underestimation in the $NO_3$-N discharge fluxes during this period. These examples clearly illustrated that while, on an average annual basis, throughfall N inputs and N outputs in the stream may be balanced at the NDW (Table 2), there were distinct periods of net N release and N retention throughout the year, and individual years did not respond uniformly in the timing or the magnitude of the N processing.

While plant uptake during the growing season undoubtedly contributed to the lower N export in the growing vs. dormant seasons, it did not explain fall and winter N export rates or the interannual and monthly variation shifts from net N retention to N release and vise versa. Uptake also could not be expected to fully control $NO_3$-N losses during the growing season considering that this was also the period of most intensive net N mineralization and nitrification with rates of internal net N release far exceeding annual N uptake rates[2,4]. Therefore, we focused on potential controls of internal N production (which occurred primarily during the growing season) vs. N transport (which was more pronounced during the dormant season) when examining the differential shifts from net N retention to N release among water years[32].

We derived simple indices of the temperature and moisture conditions during the growing season (April to September) as possible controls on microbial production and accumulation of $NO_3$-N in the soil: (1) sum of degree days >10°C and (2) total precipitation (Fig. 5). The 1990s represented considerably different temperature conditions compared to earlier decades (Fig. 2). The temperature index for the period 1990 to 1999 ranged from about 800°C (1992 and 1997) to over 1000°C (1993, 1998, 1999), compared to a cumulative temperature index of about 900°C for the remaining years. The moisture index ranged from about 800 mm (1990) to 1400 mm (1994 and 1996). There was no systematic pattern of cool-wet vs. warm-dry conditions; rather, conditions ranged from cool and wet or dry to warm and wet or dry (Fig. 5). Because of the generally udic (moist to wet) soil

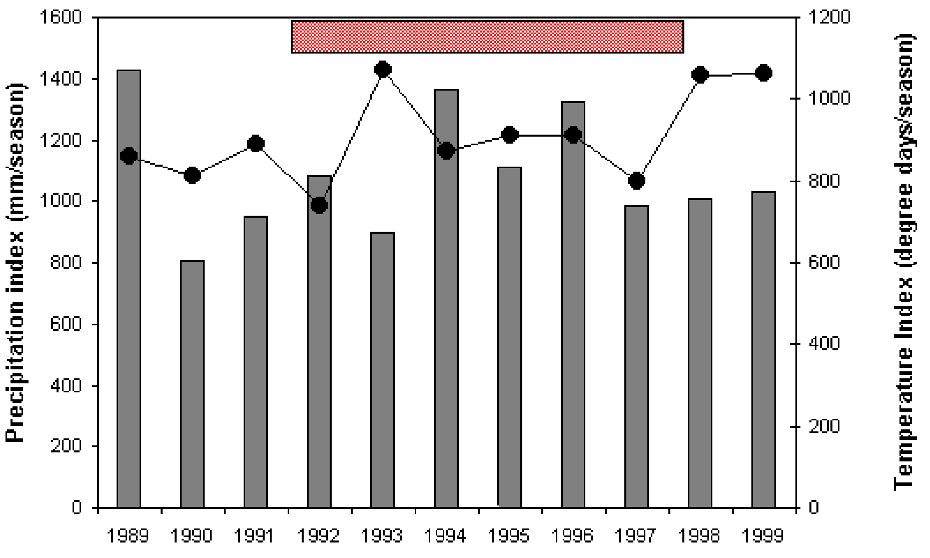

**FIGURE 5.** Time series of growing season temperature index (line graph) and moisture index (bar graph). Period for which discharge and N export data are available is indicated.

moisture conditions of this high elevation site during the growing season, N mineralization and nitrification rates would be expected to be primarily controlled by the temperature regime as demonstrated in a recent soil incubation study at the NDW showing consistently greater N release in the O and A horizons under warmer conditions[33]. Soil moisture conditions during the growing season and the subsequent fall would have a more direct control on the movement of $NO_3$-N out of the soil[34,35].

The possible implications of the diverse temperature-moisture conditions on streamwater $NO_3$-N concentrations during the fall and winter discharge period is illustrated in Fig. 6. The warm but relatively dry conditions in the summer of 1993, for example (Fig. 5), created favorable conditions for intensive nitrification and accumulation, rather than leaching, of $NO_3$-N during the growing season (shift to high net N retention in Fig. 4a). Wetter conditions in the fall following this dry period subsequently flushed out the accumulated $NO_3$-N and resulted in the elevated stream $NO_3$-N concentrations measured during the dormant period (Fig. 6, Fig. 4a). A warm but relatively wet summer, as in 1994 and 1996 (Fig. 5), still stimulated intensive nitrification, but precipitation inputs resulted in hydrologic export of part of this $NO_3$-N production during the growing season (less net N retention or even net N release and a general shift of growing season $NO_3$-N fluxes shift toward or over the 1:1 line in Fig. 4b and 4d), somewhat attenuating the $NO_3$-N flushing and the stream $NO_3$-N peaks during the wetter dormant period (Fig. 6). The lowest $NO_3$-N concentrations followed cooler summer conditions (e.g., 1992 and 1997), whether dry or more mesic, due to a temperature limitation on the microbial $NO_3$-N production process. The 1995 results were somewhat atypical as we would have expected generally higher $NO_3$-N concentrations in fall discharge based on high production and moderate hydrologic export of $NO_3$-N during the growing season. As noted earlier, missing data during the October to March period may have caused an underestimate of the peak $NO_3$-N concentrations during the dormant period. The hypothesized climatic controls on $NO_3$-N production and transport processes and the resultant effect on the magnitude and timing of $NO_3$-N export from the NDW are summarized in Table 3.

Our results agree with findings from the Solling site in Germany, which also receives high atmospheric N inputs and where nitrification and $NO_3$-N leaching pulses were observed during warm, dry years but not in wet, cold ones[36]. There are only a few long-term watershed studies under comparably high N deposition regimes against which to compare our results, and they tend to confirm our results. Production vs. transport controls on temporal streamwater $NO_3$-N signals discussed here were formulated earlier for the Turkey Lakes Watershed, Canada[32, 34,35]. Secondly, a long-term stream monitoring study in the Catskill Mountains, NY also demonstrated a distinct decoupling in timing and magnitude between atmospheric N input (~12 kg N $ha^{-1}$ $year^{-1}$) and stream N export fluxes, and concluded that microbial N transformations were the major processing step between N input and N loss with temperature rather than moisture controlling seasonal nitrification ultimately reflected in a positive correlation between stream $NO_3$-N concentrations and annual or seasonal mean air temperature[37]. Lastly, the N dynamics in the Fernow Experimental Forest, WV, which is also classified as a N-saturated forest system, exhibit many of the characteristics observed at NDW[38]. Streamwater $NO_3$-N levels under

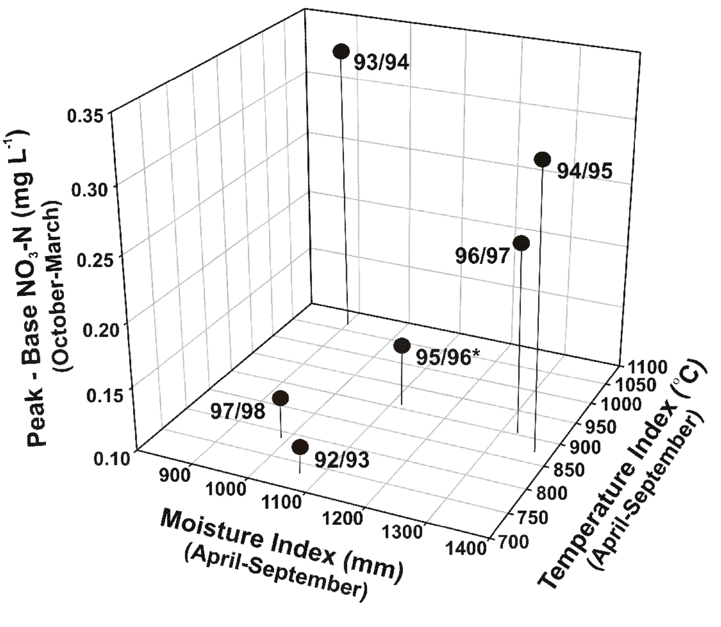

**FIGURE 6.** Hypothesized relationship between growing season moisture and temperature conditions on the dormant season $NO_3$-N concentrations (peak minus base) based on data collected during the 1992–1997 water years. *Value may be underestimated due to missing data during the October to March period.

**TABLE 3**
**Conceptual Model of Temperature and Moisture Control on the Production and Transport of $NO_3$-N in the NDW**

| *Temperature Conditions* | *Moisture Conditions* | |
|---|---|---|
| **WARM** | **DRY** | **WET** |
| Streamwater N signal | HIGH $NO_3$-N | Intermediate $NO_3$-N |
| Internal N production | High Nitrification | High Nitrification |
| Growing season N discharge | LOW | HIGH |
| Dormant season N discharge | HIGH | INTERMEDIATE |
| Example years | 1993 | 1994, 1996 |
| **COOL** | | |
| Streamwater N signal | LOW $NO_3$-N | VERY LOW $NO_3$-N |
| Internal N production | Low Nitrification | Low Nitrification |
| Growing season N discharge | LOW | INTERMEDIATE |
| Dormant season N discharge | LOW | VERY LOW |
| Example years | 1997 | 1992 |

ambient conditions have been steadily increasing over time without a similar increase in precipitation chemistry (~7 kg N $ha^{-1}$ $year^{-1}$ wet deposition for 1982 to 1993), reflecting the increasing role of internal N release via nitrification on the overall catchment N budget[38,39]. Recent studies there have further demonstrated the significance of soil moisture and ambient temperature in creating temporal variability in microbial N release and $NO_3$-N leaching while spatial variations in N processing within the watershed were more related to other ecosystem characteristics[40].

## CONCLUSIONS

The natural stand dynamics of the high-elevation spruce-fir forest in the GSMNP, coupled with recent overstory mortality due to biological and climatic disturbances, have limited the ecosystem's N retention capacity of the high atmospheric N inputs and caused the system to become N-saturated. Yet our study at the NDW has shown that even under N-saturated conditions, the catchment did not function as a simple flow-through system. Rather, significant modifications in the composition, magnitude, and timing of the N fluxes took place as water passed through the catchment, which further differed among years.

While $NH_4$-N accounted for a significant portion of the atmospheric N input flux, most of this inorganic N form was effectively retained in the forest canopy and litter layer. There was little additional modification in the $NH_4$-N solution signal suggesting a balance between the belowground $NH_4$-N sources and sinks, and $NH_4$-N generally played only a minor role in subsurface and streamwater N fluxes. Major N losses from the system occurred as $NO_3$-N, and the catchment response was more complex for this N form. While the annual N input-output budget would suggest that atmospheric $NO_3$-N simply moved through the system unaltered, our analysis showed a clear decoupling between input and output of $NO_3$-N spatially through the catchment compartments as well as in time. The modification of the hydrologic $NO_3$-N signal occurred at three levels. As was the case with $NH_4$-N, a portion of the atmospheric $NO_3$-N was retained within the forest canopy. The upper soils generally acted as a $NO_3$-N source, particularly during the active growing season, whereas deeper mineral soil layers were $NO_3$-N sinks, significantly reducing the $NO_3$-N signal prior to discharge into the stream. We suggest that it was this internal $NO_3$-N release and the climatic controls on the production and transport of $NO_3$-N that caused the lack of synchronicity between monthly N input and output fluxes as well as the interannual variation in the timing and the magnitude of the catchment $NO_3$-N export response. The dormant season generally represented a period of N loss from the catchment due to limited biological activity (lack of plant uptake) coupled to significant hydrological fluxes. The growing season was the period of intensive internal $NO_3$-N production often far in excess of plant N uptake capacity. Despite this large surge in soil solution $NO_3$-N flux, however, only a limited amount of this internal $NO_3$-N production actually ended up in the streamwater, suggesting further subsurface N retention or denitrification.

Climatic conditions during the growing season, especially temperature control on N production and hydrological control on leaching output, influenced N discharge peaks in the subsequent fall and caused the large differences in summer and fall $NO_3$-N discharge rates among different years. Greatest peak $NO_3$-N discharges would be expected in the fall period following warm (higher production) and relatively dry (limited transport) summers, with hydrologic flushing of the accumulated $NO_3$-N taking place in fall. Lower fall N discharge rates would be more characteristic following cooler (lower production) and/or wetter (nonlimited transport) summers.

Our study has clearly demonstrated the dynamic nature of the concept of N saturation as well as the need to also examine spatial and temporal N flux patterns. It should also be noted that this analysis was based only on dissolved inorganic N forms since no DON data were available. Current monitoring activities at the NDW now include analysis for DON since increasing evidence is emerging that organic N may be an important component catchment N mass balance and dynamics[41,42,43].

## ACKNOWLEDGMENTS

Data for this paper have been collected through sponsorship by the Electric Power Research Institute (EPRI) [contract RP-2621 with Martin Marietta Energy Systems Inc., operator of ORNL for the U.S. Department of Energy (USDOE)]; the USDA Forest Service Spruce-Fir Cooperative (Interagency Agreement 1494-078-A1with the USDOE); the Inventory and Monitoring Program of the National Park Service; the USDA National Research Initiative Competitive Grants Program (Grant No.97-35101-4314 to Utah State University); the USGS Biological Research Division (Cooperative Agreement No. 1434 HQ97-RV-01555 RWO34 to the Utah Cooperative Fish and Wildlife Research Unit); Tennessee Valley Authority's Public Power Institute; the Nevada Agricultural Experiment Station; and the USDOE's National Energy Technology Laboratory. We gratefully acknowledge the contributions of all those who were involved in the collection and analyses of the water samples.

| | | |
|---|---|---|
| Received: | July | 17, 2001 |
| Revised: | November | 12, 2001 |
| Accepted: | November | 12, 2001 |
| Published: | November | 22, 2001 |
